# Undetected ophthalmological disorders in Parkinson’s disease

**DOI:** 10.1007/s00415-022-11014-0

**Published:** 2022-03-09

**Authors:** Carlijn D. J. M. Borm, Mario Werkmann, Debbie de Graaf, Femke Visser, Arno Hofer, Marina Peball, Katarzyna Smilowska, Diana Putz, Klaus Seppi, Werner Poewe, Carel Hoyng, Bastiaan R. Bloem, Thomas Theelen, Nienke M. de Vries

**Affiliations:** 1 Department of Neurology, Centre of Expertise for Parkinson & Movement Disorders, Donders Institute for Brain, Cognition and Behaviour, Radboud University Medical Centre, PO Box 9101, 6500 HB Nijmegen, The Netherlands; 2 Department of Neurology, Medical University Innsbruck, Innsbruck, Austria; 3 Department of Neurology, Onze Lieve Vrouwe Gasthuis (OLVG), Amsterdam, The Netherlands; 4 Department of Ophthalmology, Medical University Innsbruck, Innsbruck, Austria; 5 Department of Ophthalmology, Radboud University Medical Centre, Nijmegen, The Netherlands

**Keywords:** Parkinson’s disease, Ophthalmology, Ophthalmological disorders, Visual impairment, Non-motor symptoms

## Abstract

**Background:**

Ophthalmological disorders are common and frequently disabling for people with Parkinson’s disease (PD). However, details on the prevalence, severity and impact of ophthalmological disorders thus far lacking. We aimed to identify PD patients with undetected ophthalmological disorders in a large cross-sectional, observational study.

**Methods:**

We previously delivered a screening questionnaire to detect ophthalmological symptoms (Visual impairment in PD questionnaire; VIPD-Q) to 848 patients. Here, we report on a subgroup of 102 patients who received complete ophthalmological assessment aimed at identifying clinically relevant ophthalmological diseases, which were classified as either vison-threatening or not. Impact on daily life functioning was measured using the visual functioning-25 questionnaire (VFQ-25) and fall frequency.

**Results:**

Almost all patients (92%) had one or more clinically relevant ophthalmological disorders. Of those, 77% had a potentially vision-threatening disease, while 34% had a potentially treatable ophthalmological disease which impacted on quality of life. The most prevalent ophthalmological disorders were dry eyes (86%), ocular misalignment (50%) and convergence insufficiency (41%). We found a weak but significant association between clinically relevant ophthalmological diseases and both fall frequency (*R*^2^ = 0.15, *p* = 0.037) and VFQ-25 score (*R*^2^ = 0.15, *p* = 0.02). The VIPD-Q could not correctly identify patients with relevant ophthalmological disorders.

**Conclusions:**

Surprisingly, in our study sample, many participants manifested previously undetected ophthalmological diseases, most of which threatened vision, impacted on daily life functioning and were amenable to treatment. Screening for these ophthalmological disorders using a questionnaire asking about symptoms seems insufficient. Instead, episodic ophthalmological assessments should be considered for PD patients, aiming to identify vision-threatening yet treatable diseases.

**Trial registration:**

Dutch Trial Registration, NL7421.

**Supplementary Information:**

The online version contains supplementary material available at 10.1007/s00415-022-11014-0.

## Introduction

Persons with Parkinson’s disease (PD) often experience ophthalmological problems, including burning eyes, visual field deficits or visual hallucinations [1, 2]. Ophthalmological symptoms are part of the non-motor symptoms of PD and are commonly observed early in the disease or even in prodromal stages [3–5]. These symptoms can result from a broad range of ophthalmological disorders, such as convergence insufficiency or decreased contrast or colour vision. Some are caused by retinal thinning and dysfunction due to retinal dopamine depletion [6–8]. Others might be age-related rather than linked to the degenerative process of PD itself, e.g. glaucoma, age-related macular degeneration or cataract [9–12]. Ophthalmological disorders, if not treated adequately, may negatively impact on physical activity, activities of daily living and quality of life [2, 13, 14]. Visual functioning seems extra important for PD patients because of their need to overcome loss of motor function with visual guidance [15]. For example, visual cueing is an established method to improve walking and reduce freezing of gait in PD, but this strategy is obviously less effective when patients cannot see the visual cues properly [16, 17].

In view of the above mentioned, ophthalmological disorders in PD have received surprisingly little interest in clinical practice and research. Earlier work compared ophthalmologic tests and ophthalmological symptoms, such as colour vision or diplopia, between PD patients and controls, but did not include a detailed search for underlying ophthalmological disorders [2, 14, 18, 19]. Therefore, little is known about the frequency, severity and impact of specific ophthalmological disorders in PD. Moreover, there is no guideline when and how to assess ophthalmological symptoms in daily practice, possibly delaying referrals to the ophthalmologist so patients are withheld effective treatments. Here, we aim to identify PD patients with undetected ophthalmological disorders, population prevalence, severity and impact of ophthalmological disorders in a convenience sample of PD patients.

## Methods

### Participants and setting

We performed a cross-sectional, observational study. The methodology has been detailed before [20]. Our initial study population consisted of a cohort of 848 PD patients who had completed the Visual Impairment in Parkinson’s Disease Questionnaire (VIPD-Q) [21]. Patients included in the present study were selected from this larger sample. Specifically, we selected candidates based on the order of submission of the VIPD-Q, until the number of 102 participants was reached. Selection bias may have been caused by the fact that we could only approach participants who had responded positively to a question in the questionnaire, asking whether we might contact the respondents for further research. In addition, the first responders may be the ones experiencing the greatest impact of ophthalmological disorders. However, our selected group showed a median of 13 points on the VIPD-Q and a range of 0–48 points. We included patients with a diagnosis of PD according to the UK Brain Bank criteria [22], age of PD onset older than 30 years, age at study inclusion of 60 years or older, and stable doses of dopaminergic replacement therapy for at least 4 weeks prior to the ophthalmological examination. Exclusion criteria included secondary causes of parkinsonism (e.g. vascular parkinsonism*)*; prior brain surgery (except deep brain stimulation); history of systemic diseases (e.g. DM type I, or type II with diabetic retinopathy), other neurodegenerative diseases; medication influencing normal visual function; prior eye surgery except uncomplicated cataract surgery; blindness in one eye (i.e. blindness according to the WHO criteria, with visual acuity worse than 3/60) [23]; and presence of dementia or major depressive or psychotic disorder according to DSM IV. Between May 2017 and May 2019, a sample of 102 patients was enrolled for an extensive neurological and ophthalmological assessment at two university hospitals specialised in movement disorders (Radboudumc, Nijmegen, the Netherlands; and Medical University, Innsbruck, Austria). The study was performed in accordance with the declaration of Helsinki. Each study centre obtained local ethical approval. All the participants gave their written informed consent prior to participation.

### Procedures and assessments

All assessments were performed while patients continued their regular medication. We obtained demographics and medical history, followed by a neurological and detailed ophthalmological assessment. For the complete list of examinations, see Supplementary 1.

### Population prevalence and severity of ophthalmological disorders

We chose a pragmatic approach, first reviewing whether people with PD had any ophthalmological disease, based on the ophthalmological exams. We then evaluated the severity of ophthalmological diseases (based on the ophthalmological assessment). None and mild disease gradation were scored as “not clinically relevant” and moderate and severe as “clinically relevant”. This classification was based on a combination of literature and expert opinion and is depicted in detail in Supplementary file 2. The definition clinically relevant is a description of the severity of an ophthalmological disorder. This is not a grade of functional disability to a patient. Corneal diseases, blepharitis or manifest ocular misalignment were categorised as either present with or without clinically relevant manifestations, or absent. Congenital colour blindness, choroidal nevus, tilted disc, vascular changes of the retina, retinal degeneration and dermatochalasis were simply classified as either present or absent. Identified ophthalmological disorders were classified as vision threatening or not, as well as potentially treatable vs not. Potentially treatable/manageable disorders are: glaucoma, dry eyes, convergence insufficiency, ocular misalignments, cataract, vitreous haemorrhage, conjunctivitis, eyelids disorders, lens capsule opacification and cornea diseases.

### Impact of ophthalmological disorders

The impact of ophthalmological disorders on daily activities was measured using the 25-item National Eye Institute Visual Function Questionnaire (VFQ-25) [24]. This questionnaire measures the influence of visual disability and visual symptoms on 11 generic health domains such as emotional well-being and social functioning, in addition to task-oriented domains related to daily visual functioning. A maximum score of 100 indicates perfect functioning in daily life. Composite scores of 10 domains of the VFQ-25 (without the domain driving) were categorised into four categories, from the highest scores to the lowest (100–81 = no impact, 80–61 = mild impact, 60–41 = moderate impact, 40–0 = severe impact) [24]. Moreover, we used fall frequency as an indicator for the impact of ophthalmological diseases on daily life activities. We asked participants how often they had experienced falls during the last 6 months preceding our study (categorised as either never, 1–2 × per month, weekly, or daily). We also explored if the VIPD-Q score could be related to quality of life.

### The Visual impairment questionnaire (VIPD-Q)

The VIPD-Q was developed to detect a broad range of ophthalmological problems in PD [20]. The questionnaire consists of 17 questions on ophthalmological symptoms, categorised into four domains of ophthalmological disorders: (1) ocular surface; (2) intra-ocular; (3) oculomotor; and (4) optic nerve. Answers were given on a 4-point Likert scale ranging from “never had symptoms” to “daily symptoms”. Our published study protocol provides detailed information on the domains included in the VIPD-Q [20].

### Statistical analysis

The baseline patient characteristics were expressed as means with standard deviation. Frequency distributions were calculated for all outcomes. Nonparametric variables were expressed as median, interquartile range (IQR) and minimum and maximum values. We compared characteristics of patients with and without vision-threatening diseases using the Student’s *t* test for parametric continuous variables and the Mann–Whitney *U* test for non-parametric continuous variables. The impact of ophthalmological diseases on daily life function (VFQ-25 total score, fall frequency) is assessed using linear regression with correction for age, Hoehn and Yahr stage, freezing of gait and disease duration. To evaluate the screening questionnaire, in terms of correctly identifying patients with ophthalmological disorders, we had originally planned to compare the results of the VIPD-Q with the outcomes of the ophthalmological assessments, using linear regression and receiver-operating characteristic (ROC) for the total score and the score per domain. We abstained from these analyses, because of the unexpectedly high prevalence of ophthalmological disorders in the PD population, making it impossible to compare patients with or without ophthalmological problems. All analyses were performed with SPSS 22.0 (SPSS Inc, IBM, Chicago, IL, USA).

### Data availability statement

Requests for data from the VIP Study will be considered by B.R.B. in line with data protection laws. The general policy is that as long as the proposed use of the data is scientifically valid and if ethics approval permits, suitably anonymised data can be shared with other researchers.

## Results

### Participants

Patient characteristics are described in Table 1. There were no differences between patients with and without vision-threatening ophthalmological diseases concerning age, sex, disease duration, Levodopa-equivalent daily dose (LEDD), Hoehn and Yahr stages, cognitive function and motor examination.

**Table 1:** Patients characteristics

Demographics^a^	PD patients (*n* = 102)
Age, median (IQR) [range], years	68 (8) [60–68]
Men, *n* (%)	69 (68)
Disease duration, median (IQR) [range], years	6 (7) [0–19]
Hoehn and Yahr stage (SD)	2 (0) [2–4]
Laterality (more affected body side), right: *n* (%)	41 (41)
Schwab & England ADL scale (SD)	84% (12)
Levodopa doses equivalent, median (IQR) [range], mg	580 (605) [0–1950]
Polypharmacy, mean (SD), number of medications	5 (3)
Education, College degree, *n* (%)	28% (28)
Country of birth (European), *n* (%)	101 (99)
Falls in the last month, *n* (%)	25 (25)
Uses visual aid, *n* (%)	99 (99)
Comorbidity	
Diabetes Mellitus type II, *n* (%)	10 (10)
Hypertension, *n* (%)	31 (30)
Cardiac arrest, *n* (%)	9 (9)
Stroke, *n* (%)	3 (3)
Rating scales	
MDS-UPDRS part III	32 (13.7)
MDS-UPDRS total score	54 (22)
Cognitive function assessment	
MoCA	27.3 (2.7)
CLOX 1	14.2 (2.6)
GDS	3.6 (2.8)
Questionnaires	
Visual function questionnaire-25 total score	82% (12.9)
VIPD-Q total score, (range)	13 (0–48)
NMSS (German version) (*n* = 72)	40 (28)
NMSS (Dutch version) (*n* = 29)	20 (5.3)
PDQ-39	23 (13.8)

For MDS-UPDRS, GDS, VIPD-Q, NMSS, and PDQ-39, higher scores indicate worse functioning

For activities of daily living scale, VFQ-25, MoCA, and CLOX, lower scores indicate worse functioning

*n* number of participants, *IQR* interquartile range, *PD* Parkinson’s disease; Levodopa Equivalent dose (LED); activities of daily living scale according to the modified Schwab and England scale (score 0–100%), *UPDRS MDS* unified Parkinson’s disease rating scale (total score 0–236), UPDRS part III (score 0–132), *MoCA* Montreal Cognitive Assessment (score 0–30); *CLOX* clock drawing test (score 0–16); *GDS* geriatric depression scale (score 0–30); *VFQ-25* visual function questionnaire-25 (score 0–100%); *VIPD-Q* Visual impairment in Parkinson’s disease questionnaire (score 0–51); *NMSS* non-motor symptoms scale (score 0–360), Dutch version (score 0–30); *PDQ-39* the Parkinson’s disease questionnaire-39 (score 0–100)

^a^Data shown as mean (SD) unless otherwise indicated

### Prevalence and severity of ophthalmological disorders

**Fig. 1:**
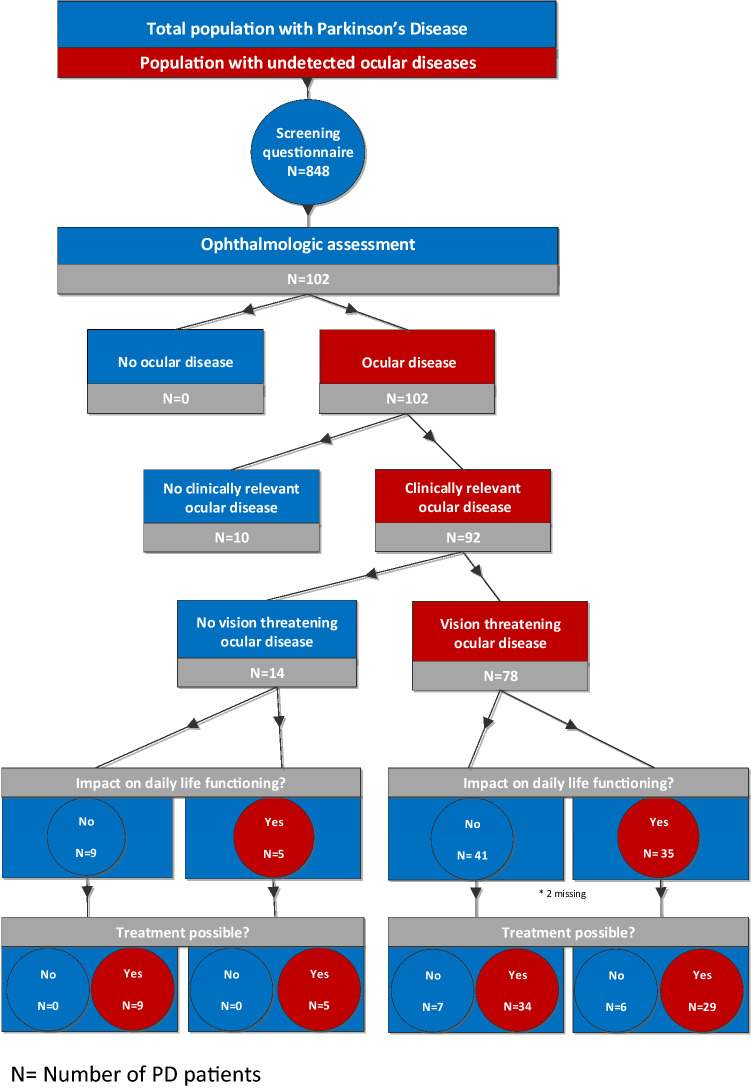
Flowchart of the number of patients with PD and ophthalmological diseases. *N* = number of PD patients

The results of the ophthalmological assessments are detailed in Table 2. The flowchart (Fig. 1) illustrates the frequency, severity, impact and treatability of the observed ophthalmological diseases. All participants had at least 1 ophthalmological disease, and 90 (92%) had an ophthalmological disease with clinical relevance; of these, 78 (77%) patients had a potentially vision-threatening ophthalmological disease. Thirty-four subjects (34%) of this subgroup had an ophthalmological disease which impacted on daily life functioning, and that could potentially be treated. Table 3 describes the prevalence of ophthalmological diseases per domain of the VIPD questionnaire. In 66% of participants, we found more than one clinically relevant ophthalmological disease, with a maximum of five different conditions. The most prevalent ophthalmological disorders were dry eyes (86%), ophthalmological misalignment (50%), optic nerve disorder (50%), convergence insufficiency (41%) and cataract (40%).

**Table 2:** Ophthalmological assessment: outcomes

Outcome	Ophthalmological Assessment ^a^	PD patients (*n* = 102)	
	Subjective ophthalmological assessment	OD mean (SD)	OS mean (SD)
Best corrected visual acuity	LogMAR value	0.06 (0.21)	0.03 (0.16)
	Snellen 20/25–20/10 (normal), %	81%	82%
	Snellen 20/32–20/63%	12%	11%
	Snellen 20/80–20/160, %	1%	2%
	Snellen worse than 20/200, %	1%	0%
	Visual acuity too low to drive (> 0.3 LogMAR)	5%	5%
Reading	Reading speed, wpm, M:5	165 (49)	
	Reading speed, wpm, M:0.25 (n = 12)	73 (32)	
	Near visual acuity (LogMAR value)	0.14 (.-0.20-0.50)	
	Snellen 20/25–20/10 (normal), %	60%	
	Snellen 20/32–20/63,%	39%	
	Snellen 20/80–20/160, %	1%	
Visual field	Humphrey field analyzer, MD (n = 30)	− 4.35 (4.6)	− 4.7 (5.5)
	Octopus, MD (n = 71)	5.3 (4.6)	6.1 (4.8)
	Metamorphopsia	13%	18%
	Amsler grid (cannot see all corners/sides)	7%	8%
	Visual field deficit (moderate/severe)	43%	50%
Contrast vision	Low-contrast letter charts (Pelli-Robson), CSS	1.53 (0.20)	1.52 (0.20)
	Moderate/severe decreased (CSS < 1.50)	40%	50%
Colour vision	Ishihara plates (range)	18 (1–21)	18 (1–21)
	Farnsworth Munsell hue test (desaturated 15D)		
	Mild green blind	2%	6%
	Moderate/severe green blind	3%	3%
	Mild blue blind	48%	47%
	Moderate/severe blue blind	24%	26%
	Mild red blind	2%	4%
	Moderate/severe red blind	5%	6%
Convergence	Near point of convergence, cm	10.97 (0.28)	

	Objective ophthalmological assessment	OD mean (SD)	OS mean (SD)
Cataract grading Lens opacity (LOCS III)	Pseudophakia	20%	19%
	NO (score 4 >)	4%	4%
	NC (score 4 >)	4%	4%
	Cortical (score 3 >)	13%	14%
	Subcapsular (score 3 >)	2%	0%
Corneal thickness	Pachymetry, µm	538.14 (48.84)	533.75 (56.09)
Cornea appearance	Keratitis punctate/dry cornea	10% (10)	11% (11)
	Cornea erosion/scarring	3% (3)	2% (2)
Intra-ocular pressure	(Goldman) tonometry, mmHg	14 (3.04)	14 (2.76)
Tear production	Schirmer’s II test, mm	9.86 (7.4)	9.17 (4.31)
	TFBUT, s	9.0 (4.31)	9.06 (4.37)
	Eye blink rate, blinks/minute	15 (12.75)	
Eyelids	Loss of lashes	1%	1%
	Eye lid retraction	1%	1%
Conjunctiva	Hyperaemia	4%	5%

*OD* oculus dextra, *OS* oculus sinistra

*BCVA* best corrected visual acuity, in LogMAR. Normal value ≤ 0.10 LogMAR, or ≥ 20/25 Snellen visual acuity. Reading is assessed with the Radner reading chart. *Wpm* words per minute. Visual field is tested with the Humphrey and Octopus Automated Field Analyser in a standardised design. The mean deviation (MD) is notated. Lower MD score indicates for the Humphrey worse visual field deficit, normal value MD > 0. Higher MD score indicates for the Octopus worse visual field deficit, normal value MD ≤ -0.8

Pelli–Robson assessment consists of letters arranged in groups with varying contrast, from high to low, calculated in the CSS: contrast sensitivity score, (score 0–2.25)

To evaluate colour vision pseudo-isochromatic plates with coloured dots forming numbers (Ishihara plates) are used. Farnsworth desaturated 15D hue test is performed to evaluate subtler colour vision deficiencies. This task consists of ordering 15 coloured caps over trays in an incremental manner according to their hue

*NPC* near point of convergence in centimetre (cm)

To detect cataract the lens opacity is rated with the LOCSIII, Lens opacities classification system; *NO* nuclear opalescence (score 1–6), *NC* nuclear colour (score 1–6), Cortical (score 1–5), Posterior (score 1–5). Scores noted in table represent clinically relevant cataract

Cornea, eyelids and cornea are inspected. *µm* micrometre; *IOP* intra-ophthalmological pressure measured with applanation tonometry. Tear film quality is approached by the Tear-Film-Break-Up-Time (TFBUT), while the quantity of tears is measured by the Schirmer test. Schirmer II test, paper strips are inserted into the lower fornix with local anaesthesia, wet distance is measured in millimetres (mm), *EBR* eye blink rate is measured in blinks/minute

For NPC, LOCsIII and IOP higher scores indicate worse outcome

For visual acuity, CSS, Schirmer II, TFBUT and number of Ishihara plates lower scores indicate worse outcome

^a^Data shown as mean (SD) unless otherwise indicated

**Table 3:** Prevalence of ophthalmological disorders

PD patients, age 60–86, *n* = 102	OD (%)	OS (%)
Ocular surface		
Cornea disease ^a^	6	6
Blepharitis	2	2
Dermatochalasis	13	14
Conjunctivitis sicca (mild-severe)	86	86
Conjunctivitis sicca (moderate/severe)	27	27
Conjunctivitis	4	5
Oculomotor		
Experiencing diplopia*	30	
Convergence insufficiency	41	41
Ocular misalignment	54	50
Manifest	5	6
Intra-ocular		
Cataract^b^	17	18
Lens capsule opacification	3	2
Pseudophakia	20	19
Vitreous haemorrhage	0	3
Retina/optic nerve		
Maculopathy ^c^	27	22
AMD dry	24	20
AMD wet	0	0
Diabetic macular oedema	0	0
Optic nerve disorder ^d^ (*n* = 85/85)	50	52
Glaucoma suspect ^e^	20	15
Elevated IOP	1	1
Retinopathy ^f^	2	1
Retinal vascular changes	23	28
Peripheral drusen	10	9
Additional		
Nevus choroid	7	3
Tilted disc	7	4
Congenital colour blindness ^g^	5	
Visual Hallucinations	18	
Amblyopia	3	

*OD* oculus dextra, *OS* oculus sinistra

*Based on the VIPD-Q questionnaire. Not specifically during reading

^a^Group of cornea diseases, consists of: cornea scar, verticillate, dystrophy (punctate/erosions are part of the diagnosis of dry eyes),

^b^Cataract clinically significant, total percentage OD with cataract (present of historic):40%, OS: 39%

^c^Group of macular diseases, consists of: AMD (age-related macular degeneration), scar, epiretinal gliosis, telangiectasia, macular oedema,

^d^Group of optic nerve disorders consists of: optic nerve head drusen, optic nerve head atrophy, optic nerve head pallor

^e^Glaucoma suspects, CDR (cup-to-disc-ratio) > 0.5 and a typical visual field deficit, *IOP* intra-ophthalmological-pressure

^f^Group of retinopathies consists of: branch retinal vein occlusion, blot bleeding,

^g^In the study, population congenital colour blindness occurred in 7% of the men and 0% woman

### Impact of ophthalmological diseases on daily life functioning and falling

**Fig. 2:**
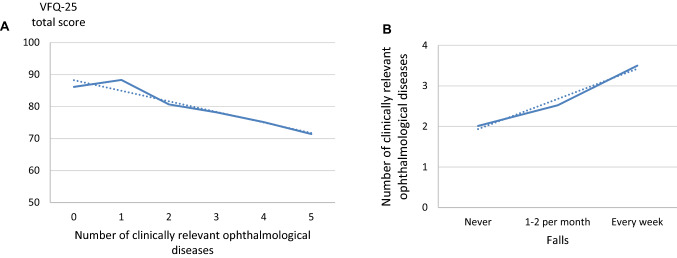
Impact of ophthalmological diseases. **A** Impact of clinically relevant ophthalmological diseases (grade moderate to severe) on the visual functioning-25 questionnaire (VFQ-25). A total score of 100 on the VFQ-25 indicates perfect functioning in daily life, lower scores relate to impaired function due to ophthalmological symptoms. Shown here is a decrease in the VFQ-25 total score with an increase in ophthalmological diseases. **B** The impact of ophthalmological diseases on the frequency of falls in the last half year. Patients tend to fall more often with an increased number of clinically relevant ophthalmological diseases

Ophthalmological diseases in PD patients impacted daily life functioning, as shown by the VFQ-25. Specifically, the mean total score of the VFQ-25 was 82% ± 12.9. Almost half of the PD patients (43%) reported functioning under the 80% threshold, indicating a negative effect on daily life functioning. The scores per domain are shown in Table 4. We found a weak but significant association between the VFQ-25 score and the number of clinically relevant ophthalmological diseases (*R*^2^ = 0.15, *F*_3,93_ = 5.165, *p* = 0.002, corrected for age and disease duration, Fig. 2A). In addition, a higher fall frequency was associated with a larger number of clinically relevant ophthalmological diseases (*R*^2^ = 0.15 *F*_5,91_ = 3.12, *p* = 0.037, corrected for age, Hoehn and Yahr stage, freezing of gait and disease duration, Fig. 2B).

**Table 4:** Visual function questionnaire-25 scores

VFQ Sub-Scale	PD patients *n* = 102
	(Mean ± SD)
Composite score **	82 ± 13
Role difficulties	68 ± 28
General vision	77 ± 21
Ocular pain	78 ± 17
Driving*	78 ± 18
Vision specific mental health	81 ± 20
Near activities	82 ± 17
Distance activities	82 ± 17
Peripheral vision	88 ± 17
Vision specific social functioning	90 ± 14
Vision specific dependency	91 ± 20
Colour vision	94 ± 11
General health	68 ± 16

Subscale ordered highest to lowest impact

*NEI VFQ-25* 25-item National Eye Institute Visual Function Questionnaire 10 (maximum score, 100), *SD* = standard deviation

*39 subjects do not drive and were excluded from the driving subscale

**Composite score without driving

## Discussion

In our study, ophthalmological disorders were common and debilitating among our sample of PD patients aged 60 years and older. Almost all people with PD had at least one clinically relevant ophthalmological disease (92%), and almost half of them (44%) reported impaired daily functioning because of these ophthalmological disorders. Importantly, many of these ophthalmological disorders were potentially treatable.

### Population prevalence and severity of ophthalmological disorders

The high population prevalence of multiple ophthalmological diseases in our sample is noteworthy, particularly because little attention is typically paid to this issue in daily practice [25]. In the general population, approximately 80% of all causes of visual impairment are considered to be avoidable. Once a correct diagnosis has been established, many effective treatments are available [26]. Our present findings indicate that effective treatments are also available for many ophthalmological diseases in PD, especially dry eyes and cataract. Therefore, ophthalmological screening of PD patients is highly advisable.

From this analysis, keratoconjunctivitis sicca (dry eyes) was the most prevalent ophthalmological disease found in our PD population. Dry eyes was found in 86% of participants, which is much higher than reported previously (60%) [2, 14, 27]. This may be the result of our relatively old study population. Various factors may contribute to dry eyes in PD, including reduced eye blink rate, dysfunction of the sebaceous Meibomian glands, autonomic dysfunction and blepharitis [28–30]. Timely diagnosis and optimal treatment of dry eyes, e.g. with artificial tears, may increase the optical quality of the cornea and therefore improve the visual quality. Because of the high prevalence, standard treatment of patients with PD of 60 years and older with artificial tears could be considered.

Half of our study population had oculomotor deficits, which is a well-described feature in PD [31, 32]. Latent ocular misalignment or heterophoria, which is a subtle manifestation that can attribute to diplopia, was most frequent. While this has been described as an age-related symptom, we found a much higher prevalence in PD patients (50%) as compared to a healthy elderly population in the literature (15%) [33]. Earlier work shows that convergence insufficiency often contributes to diplopia [34]. We found convergence insufficiency in 41% of our population. Convergence ability deteriorates with age. However, it was more prevalent among PD patients than controls, both in on and off medication state [34, 35]. This suggests that ophthalmological fusional mechanisms are particularly vulnerable in PD. However, these problems can be effectively treated, for example with a prism or occupational therapy [36].

Retinal and optic nerve pathology was present in 30–50% of our study population. We found atrophy of the optic nerve head accompanied by optic nerve head pallor in half of the patients, mostly in the temporal quadrants. Optic nerve changes in PD are likely caused by primary neurodegeneration [37]. The retina and optic nerve head have drawn a lot of interest since the introduction of optical coherence tomography (OCT). The pattern of thinning of the retina and visual field deficit in PD seems to mimic that of glaucoma, with a relative sparing of the fibres entering the optic nerve head nasally [38–41]. Our data showed a much higher rate of possible glaucoma cases (17.5%) than may be expected for the general population (3.5%) [42]. Therefore we hypothesise that PD-related optic neurodegeneration may clinically mimic (normal pressure) glaucoma. In our cohort, there was only one patient with an increased intra-ophthalmological pressure. Previous studies reported a higher incidence of open-angle glaucoma in PD patients, suggesting a shared neurodegenerative process [11, 14, 43]. However, there is currently no reason to assume a significantly higher glaucoma risk for PD patients compared to healthy controls. We tried to evaluate if participants with optic nerve atrophy scored lower on the Visual Functioning questionnaire, indicating a disability in daily life functioning, however, this was not possible since almost all patient have next to an optic nerve atrophy a different ophthalmological disorder. Further research here is warranted which, to prevent possible overinterpretation and maybe unnecessary treatment of pseudo-glaucoma in PD.

Maculopathy was present in a quarter of our patients, with the most common cause being age-related macular degeneration (AMD). Only one other study reported the prevalence of AMD in PD, which was lower compared to our data [14], possibly because of a younger patient cohort. Intriguingly, two studies reported an increased risk of a new diagnosis of PD when AMD was previously diagnosed [9, 10]. We speculate that the underlying pathology of both diseases may contain some similarities, although the only known overlapping factor is an increase of prevalence with ageing [10].

### Impact of ophthalmological disorders on daily life functioning

Non-motor symptoms such as ophthalmological disorders can be as debilitating as motor symptoms in PD and contribute significantly to a poor quality of life [31, 44]. In our study, 44% of patients with relevant ophthalmological diseases reported that their visual disability influenced their daily functioning negatively (mean VFQ composite score 82). This is also reflected by the correlation between a lower VFQ total score and a higher number of clinically relevant ophthalmological disorders. Even though this correlation was weak, the combined results indicate that vision-related quality of life may be worse in PD subjects with more clinically relevant ophthalmological diseases. The most impaired subscales of the VFQ-25 were ocular pain, general vision, near visual activities, and peripheral vision. These findings are consistent with our results of the most common ophthalmological disorders. For example, dry eyes may cause ocular pain and general vision problems. Only one small earlier study, studied vision-related quality of life in PD with the VFQ-25. A worse composite score was found in PD patients compared to controls (VFQ composite score of 96 *n* = 16 vs 87, *n* = 27) [35]. Unfortunately, this study did not consider dry eyes as a contributor for impaired quality of life. Other studies on vision-related quality of life focussed on the impact of single ophthalmological symptoms, such as contrast sensitivity or visuospatial disruption. They found that these contributed to difficulties performing daily life activities, such as driving, walking and reading [1, 45, 46].

Ophthalmological diseases might contribute to frequent falling and necessitate reductions in physical activity. Established risk factors for falling in PD mainly include motor features, such as freezing of gait or balance impairment [47]. The possible contribution of non-motor symptoms, such as ophthalmological problems, has been studied in much less detail. Among our patients, 25% reported at least one fall in the last 6 months. A higher fall frequency was associated with a greater number of clinically relevant ophthalmological diseases, even when corrected for age, disease duration, freezing of gait and Hoehn and Yahr stage. This suggests that patients with an accumulation of clinically relevant ophthalmological diseases tend to fall more often. Since recurrent falls are considered to be a clinical milestone in PD that increase the risk of e.g. nursing home admission and mortality, this is an important finding [15], particularly because several ophthalmological disorders can be treated, thus helping to prevent at least some of the future falls. Nevertheless, our findings should be interpreted with caution, since the group of fallers in this study was small (*n* = 25) and also because the effect size is relatively small, which may lead to an overestimation of the outcome. Moreover, we did not specify the reason of falling and there is a potential recall bias. The key point is that ophthalmological issues should be considered among the many different factors that jointly contribute to the risk of falls. Pending further evidence, we do recommend that frequent fallers should be thoroughly screened for presence of ophthalmological disorders, using a detailed ophthalmologic examination focussing in particular on the common ophthalmological disorders identified here. Future intervention study should demonstrate whether adequate treatment of any identified ophthalmological disorders will help to reduce the risk of falls and enhance the independency of PD patients.

### Value of a screening questionnaire

Since all ophthalmological diseases that we detected were potentially relevant and since all could have justified a referral to an ophthalmologist, the VIPD-Q in its current form does not seem to be of additional value to initiate an effective referral for ophthalmologic screening. Every patient had at least one ophthalmological disease, even those patients who scored zero points on the VIPD-Q (indicating no ophthalmological symptoms). Therefore, it was not possible to find a cutoff point to identify those patients with more than one ophthalmological disorder. This finding may be explained by several factors. First, patients may be unaware of ophthalmological symptoms. Some ophthalmological diseases have an asymptomatic progression until a very late stage (e.g. glaucoma) [48]. In older adults, visual impairment is often unrecognised, because visual changes can be relatively subtle, progress slowly over time, or occur in persons with concurrent cognitive dysfunction [48]. Therefore, actual ophthalmological disease may be difficult to screen using a questionnaire on problems. Moreover, patients may focus in particular on their motor symptoms without paying attention to their sight, so many ophthalmological problems are not volunteered during routine clinical consultations [49].

### Limitations

Our study had certain limitations. First, because we did not have a control group, it is unclear if the observed prevalence rates of specific ophthalmological disorders for PD patients are truly different from the general population. The prevalence numbers observed here are specific to our present study population, and we do not know if these are generalizable to the complete population of people with PD. As such, our current findings can only be interpreted as a first indication that relevant ophthalmological problems may be commonly overlooked in daily clinical practice. Determining the true prevalence of this issue requires further study.

In the ophthalmologic literature, prevalence data are mostly categorised for specific age groups; therefore, it was difficult to compare the population prevalence of the ophthalmological disorders in our cohort to those reported in the literature. We acknowledge that selection bias may have been caused by the fact the first responders may be the ones experiencing the greatest impact of ophthalmological disorders, although the scores on the VIPD-Q showed a large range of 0–48 with a median of 13 points and the median score was 10 (range 0–48) in the total group of 848 responders. Second, we planned to compare patients with and without ophthalmological disorders, but the group without an ophthalmological disease was very small. Therefore, a comparison was not possible. A third limitation is the challenge to replicate a normal clinical ophthalmological practice within a research setting. We tried to minimise this using a strict protocol and expert assessment.

### Future perspective

Further efforts are needed to develop improved screening tools to timely detect (and subsequently treat) ophthalmological disorders in PD patients, before they deteriorate further and begin to impact on daily functioning. One possibility is to improve the present VIPD-Q, for example by adding questions about the severity of ophthalmological problems. Until more reliable screening tools are available, we recommend that clinicians consider an episodic routine screening of people with PD by an ophthalmologist. However, we did not study the possible effects of routine screening by an ophthalmologist. Future research should study these effects, in addition to focussing on identifying the optimal moment, interval rate (ophthalmological disorders may become more prevalent as PD progresses and as patients grow older), and method of screening.

Of other great interest is the optic nerve atrophy and possible retinal thinning pattern. Recently, several studies have focussed on this issue [38–41]. Parkinson patients show an optic nerve atrophy and retinal thinning pattern, which resembles the retinal patterns in glaucoma patients and different neurodegenerative diseases, like Alzheimer’s disease. The question remains if this pattern could play a role as a possible biomarker. To solve that question, in depth longitudinal analyses are needed, with detailed clinical descriptions of the separate syndromes, which should dictate future research on this topic.

## Supplementary Information

Below is the link to the electronic supplementary material.

Supplementary file1 (DOCX 61 KB)

Supplementary file2 (DOCX 54 KB)
