# Supplementary material for: Undetected ophthalmological disorders in Parkinson’s disease
Source: J Neurol. 2022 Mar 9;269(7):3821–32. doi: 10.1007/s00415-022-11014-0 (PMC9217779; doi:10.1007/s00415-022-11014-0)
Supplement: Supplementary file 1 — Supplementary file1 (DOCX 61 KB) [file 415_2022_11014_MOESM1_ESM.docx]

**Supplementary 1**

| **Outcome** | **Ophthalmological Assessment** |
| --- | --- |
|  | *Subjective ophthalmological assessment* |
| Visual acuity | ETDRS logMAR |
| Reading speed, near visual acuity | Radner Reading Chart |
| Visual fields | Humphrey field analyser 30-2/ Octopus field analyser , Amsler grid |
| Contrast vision | Low-contrast letter charts (Pelli-Robson), |
| Colour vision | Ishihara plates / Farnsworth Munsell hue test (desaturated 15D) |
| Ocular motility | Eye follow movements, Cover test 30cm and 6 meter, Prism test (15 diopter), Eye-Tracking |
| Facial emotion recognition | Emotion Recognition Inventory (ERI) |
| Visual function | VFQ-25, Visual impairment screening questionnaire |
|  | *Objective ophthalmological assessment* |
| Lens opacity | Slit lamp examination |
| Fundus appearance | Color Fundusphotography (7-fields) |
| Corneal thickness | Pachymetry |
| Intraocular pressure | (Goldmann) tonometry |
| Tear production | Schirmer’s test, TearFilmBreakUpTime, eye blink rate |
| Anatomical changes of the retina including thickness | Spectral-domain OCT (Heidelberg) |
| Retinal micro vascular changes | Angio-OCT (Heidelberg) |
| **Outcome** | **Neurological assessment** |
| PD-related disability and impairment | MDS-UPDRS, Schwab & England activities of Daily living, NMSS, PDQ-39. |
| Gait and Balance | Timed Up and Go test, Single Leg Stance test, Gait Speed (4 meters walking), Dual tasking, Freezing of Gait, Tandem gait |
| Hand-eye coordination | 9 Hole Pegboard test |
| Cognitive function | MoCA, CLOX |
| Mood | GDS |

List of study protocol assessments, divided in ophthalmological and neurological assessment
Abbreviations: EDTRS, Early Treatment of Diabetic Retinopathy Study , D, diopter, OCT, optical coherence tomography, NMSS, non-motor symptoms assessment scale, PDQ-39, Parkinson’s Disease Questionnaire, MoCA [47], Montreal Cognitive Assessment, CLOX, clock drawing test [48], GDS, Geriatric depression scale [49], VFQ-25, visual function questionnaire[1, 2]

*Neurological examination*PD symptoms and severity including non-motor symptoms are measured using the Movement Disorders Society Unified Parkinson’s disease Rating Scale (MDS-UPDRS) [3]. The non-motor symptoms scale (NMSS) [4] and the Parkinson’s disease questionnaire-39 (PDQ-39- quality of life) Hand-eye coordination and manual dexterity are tested using the nine-hole peg test [5]. Mood and cognition are assessed by the Montreal cognitive assessment (MoCA), the CLOX II test and the geriatric depression scale (GDS).

*Gait and Balance*
Performance tests measuring different aspects of gait and balance were performed. This included walking velocity (under single and dual-tasking conditions [6, 7]), the Timed-Up-and-Go-test (TUG) [8], freezing of Gait (FOG) (assessed by letting the patient make a 360° turn with small steps (approximately 25% of their own preferred step length) and walking fast with short stepsSnijders, Nijkrake [9]), the tandem gait [10], the Single Leg Stance test (SLS)[10-13], and postural stability (recorded by the Pull-Test (item 3.12 MDS-UPDRS)).

Ophthalmological assessment
*Subjective assessment*
The best corrected visual acuity at distance was assessed, by using the Early Treatment of Diabetic Retinopathy Study (ETDRS) chart at 6 meters [14]. 20/30- 20/60 is considered mild vision loss, or near-normal vision, 20/70- 20/160, moderate visual impairment and 20/200-20/400 severe visual impairment. This was followed by testing reading or near visual acuity, tested with the Radner reading charts at 40 cm distance [15]. Possible visual fields deficits including problems in the peripheral vision were tested with the Humphrey or Octopus Automated Field Analyzer in a standardized design (SITA STANDARD 30-2) and analysed by an ophthalmologist[16]. The ability to detect objects at low contrast (contrast sensitivity), was measured contrast with the Pelli-Robson charts. This assessment consisted of letters arranged in groups with varying contrast, from high to low. Scores are based on the contrast of the last group in which two or three letters were correctly read, this can be calculated in a logarithmic contrast sensitivity score (CSS). In an elderly population (above 60 years old) a CSS lower than 1.50 is considered as decreased contrast sensitivity[17]. Colour discrimination is the ability to distinguish differences between shades of colours and is divided in primary colours (green, red, blue and yellow) and their axis (red-green, blue-yellow). To evaluate colour vision pseudo-isochromatic plates with coloured dots forming numbers (Ishihara plates) were used as a screening tool. Completed by the Farnsworth desaturated 15D hue test to evaluate subtle colour vision deficiencies [18, 19].
Ocular motility evaluation was performed by an orthoptist. Eye follow movements were used, to detect any gaze paresis, nystagmus or saccadic intrusions. To detect latent or manifest misalignment we used the alternate-cover test at 30cm and 6 meter distance [20] . Additionally, convergence insufficiency was evaluated, measuring the near point of convergence (NPC), convergence amplitudes and an exodeviation at near. Healthy (young) individuals can avoid double vision until 6cm in front of their nose. A NPC value that is more than 10 cm from the bridge of the nose is considered abnormal.

*Objective assessment*
First, we examined the eyelids and conjunctiva, followed by inspection of the cornea, iris using slit lamp examination. This to detect for example blepharitis or conjunctivitis. Lens opacity was rated with the LOCSIII score, this is used to grade cataract [21] (a LOCS score of 4≥ for nuclear cortical cataract was considered clinical relevant and a LOCS of 3≥ for capsular/postcapsular). After pupil dilation with tropicamide 0,5% fundus photography was captured. The same experienced ophthalmologist evaluated all fundus images to detect pathology of the optic nerve head, cup to disc ratio (CDR), retina, macula and peripheral area including vascular structures. A CDR greater than 0.5 with typical visual field deficits was diagnosed as glaucoma, taking intra ocular pressure and cornea thickness in account. An eye pressure higher than 18mmhg is considered higher than normal and has an increased risk for glaucomatous damage of the optic disc [22].
Diagnosis of ocular surface diseases were divided in keratoconjunctivitis sicca, cornea clouding. To establish this the criteria from (REF) were used. TearFilmBreakUpTime (TFBUT) and Schirmer test were used. TFBUT is recorded as the time between a complete blink and the appearance of the first randomly distributed dry spot, where a score less than 10 seconds is considered abnormal [23]. The Schirmer test measures the amount of fluid appearing on the ocular surface within 5 minutes. For this, paper strips are inserted into the lower fornix with (Schirmer II) local anaesthesia, and the wet distance is measured in millimetres. Less than 10 mm is considered abnormal and less than 5 is severe [23-25].

In Parkinson patients, a reduced eye blink rate EBR may add to an ocular surface disease and dry eye symptoms The EBR is defined as the number of eye blinks per minute measured in a 3-minute interval, less than 15 blinks per minute is considered abnormal [25, 26]. Finally, optical coherence tomography (OCT) will be performed to evaluate pattern changes of the retina including the retinal thickness [27-29]. Angio-OCT will be used to inspect the micro vascular changes of the retina [30].

**References**

1. Mangione, C.M., et al., *Development of the 25-item National Eye Institute Visual Function Questionnaire.* Arch Ophthalmol, 2001. **119**(7): p. 1050-8.

2. Santaella, R.M. and F.W. Fraunfelder, *Ocular adverse effects associated with systemic medications : recognition and management.* Drugs, 2007. **67**(1): p. 75-93.

3. Goetz, C.G., et al., *Movement Disorder Society-sponsored revision of the Unified Parkinson's Disease Rating Scale (MDS-UPDRS): scale presentation and clinimetric testing results.* Mov Disord, 2008. **23**(15): p. 2129-70.

4. Chaudhuri, K.R., et al., *The nondeclaration of nonmotor symptoms of Parkinson's disease to health care professionals: an international study using the nonmotor symptoms questionnaire.* Mov Disord, 2010. **25**(6): p. 704-9.

5. Ruzicka, E., et al., *Tests of manual dexterity and speed in Parkinson's disease: Not all measure the same.* Parkinsonism Relat Disord, 2016. **28**: p. 118-23.

6. Bootsma- van der Wiel, A., et al., *Single versus dual task walking performance as predictor of falls in the general population of oldest old. results of the Leiden 85-plus Study.* J Am Geriatr Soc, 2003. **51**: p. 1466-1471.

7. Mak, M.K. and M.Y. Pang, *Parkinsonian single fallers versus recurrent fallers: different fall characteristics and clinical features.* J Neurol, 2010. **257**(9): p. 1543-51.

8. Mak, M.K. and M.Y. Pang, *Balance confidence and functional mobility are independently associated with falls in people with Parkinson's disease.* J Neurol, 2009. **256**(5): p. 742-9.

9. Snijders, A.H., et al., *Clinimetrics of freezing of gait.* Mov Disord, 2008. **23 Suppl 2**: p. S468-74.

10. Borm, C., et al., *Axial motor clues to identify atypical parkinsonism: A multicentre European cohort study.* Parkinsonism Relat Disord, 2018.

11. Jacobs, J.V., et al., *Multiple balance tests improve the assessment of postural stability in subjects with Parkinson's disease.* J Neurol Neurosurg Psychiatry, 2006. **77**(3): p. 322-6.

12. Abdo, W.F., et al., *Ten steps to identify atypical parkinsonism.* J Neurol Neurosurg Psychiatry, 2006. **77**(12): p. 1367-9.

13. Aerts, M.B., et al., *Ancillary investigations to diagnose parkinsonism: a prospective clinical study.* J Neurol, 2015. **262**(2): p. 346-56.

14. Kinyoun, J., et al., *Detection of diabetic macular edema. Ophthalmoscopy versus photography--Early Treatment Diabetic Retinopathy Study Report Number 5. The ETDRS Research Group.* Ophthalmology, 1989. **96**(6): p. 746-50; discussion 750-1.

15. Maaijwee, K., et al., *Reliability testing of the Dutch version of the Radner Reading Charts.* Optom Vis Sci, 2008. **85**(5): p. 353-8.

16. Landers, J., et al., *Comparison of visual field sensitivities between the Medmont automated perimeter and the Humphrey field analyser.* Clin Exp Ophthalmol, 2010. **38**(3): p. 273-6.

17. Mantyjarvi, M. and T. Laitinen, *Normal values for the Pelli-Robson contrast sensitivity test.* J Cataract Refract Surg, 2001. **27**(2): p. 261-6.

18. Farnsworth, D., *Testing for color deficiency in industry.* AMA Arch Ind Health, 1957. **16**(2): p. 100-3.

19. Hardy, L.H., *Standard illuminants in relation to color-testing procedures.* Arch Ophthal, 1945. **34**: p. 278-82.

20. Thomas, R., A. Braganza, and T. George, *Practical approach to diagnosis of strabismus.* Indian J Ophthalmol, 1996. **44**(2): p. 103-12.

21. Chylack, L.T., Jr., et al., *The Lens Opacities Classification System III. The Longitudinal Study of Cataract Study Group.* Arch Ophthalmol, 1993. **111**(6): p. 831-6.

22. Tanito, M., et al., *Correlation between intraocular pressure level and optic disc changes in high-tension glaucoma suspects.* Ophthalmology, 2003. **110**(5): p. 915-21.

23. Milner, M.S., et al., *Dysfunctional tear syndrome: dry eye disease and associated tear film disorders - new strategies for diagnosis and treatment.* Curr Opin Ophthalmol, 2017. **27 Suppl 1**: p. 3-47.

24. Kwon, O.Y., et al., *Schrimer test in Parkinson's disease.* J Korean Med Sci, 1994. **9**(3): p. 239-42.

25. Tamer, C., et al., *Tear film tests in Parkinson's disease patients.* Ophthalmology, 2005. **112**(10): p. 1795.

26. Zaman, M.L. and M.J. Doughty, *Some methodological issues in the assessment of the spontaneous eyeblink frequency in man.* Ophthalmic Physiol Opt, 1997. **17**(5): p. 421-32.

27. Aaker, G.D., et al., *Detection of retinal changes in Parkinson's disease with spectral-domain optical coherence tomography.* Clin Ophthalmol, 2010. **4**: p. 1427-32.

28. Mailankody, P., et al., *Optical coherence tomography as a tool to evaluate retinal changes in Parkinson's disease.* Parkinsonism Relat Disord, 2015. **21**(10): p. 1164-9.

29. Ucak, T., et al., *Analysis of the retinal nerve fiber and ganglion cell - Inner plexiform layer by optical coherence tomography in Parkinson's patients.* Parkinsonism Relat Disord, 2016. **31**: p. 59-64.

30. de Carlo, T.E., et al., *A review of optical coherence tomography angiography (OCTA).* Int J Retina Vitreous, 2015. **1**: p. 5.
