# Supplementary material for: Undetected ophthalmological disorders in Parkinson’s disease
Source: J Neurol. 2022 Mar 9;269(7):3821–32. doi: 10.1007/s00415-022-11014-0 (PMC9217779; doi:10.1007/s00415-022-11014-0)
Supplement: Supplementary file 2 — Supplementary file2 (DOCX 54 KB) [file 415_2022_11014_MOESM2_ESM.docx]

**Supplementary file 2: Table, gradation of ocular disorders or problems divided in four domains of ocular anatomy.**

| **Ocular disorders/ problem** | **Test** | **Gradation** | | | | **Explanation** | **Reference** |
| --- | --- | --- | --- | --- | --- | --- | --- |
|  |  | **None** | **Mild** | **Moderate** | **Severe** |  |  |
| Visual acuity | EDTRS charts  LogMAR | -0.10 to 0.10 | 0.20-0.50 | 0.60-0.90 | 1.00 ≥ | 0.3 is minimum for driver’s license | [1, 2] |
| **Ocular surface** |  | **None** | **Mild** | **Moderate** | **Severe** |  |  |
| Eye lids disorders | Inspection | Not present | x | Present | | Blepharitis, dermatochalasis, blepharospasm | Expert assessment* |
| Conjunctival disorders | Inspection | Not present | x | Present | | Conjunctivitis, hyperaemia | Expert assessment |
| Cornea disease | Inspection  Slit lamp | Not present | x | Present | | Scar, verticillate, dystrophy, erosion, punctate | Expert assessment |
| Conjunctivitis sicca | Symptoms and  Clinical signs and | Not present | Level1/  level 2 | Level 3 | Level 4 | Levels of severity of DTS without lid margin disease according to symptoms and signs. | [3] [4] |
|  | TFBUT and | ≥ 10 seconds | x | < 10 seconds | |  | [4-6] |
|  | Schirmer II * | ≥ 10 mm | 5- 10 mm | | < 5 mm | *after applying local anaesthesia | [4-6] |
| **Oculomotor disorders** |  | **None** | **Mild** | **Moderate** | **Severe** |  |  |
| Convergence insufficiency | Near Point of Convergence | ≤10 cm* | 10-15 cm | 15-20 cm | 20 cm ≥ | *Normal values for age ≥ 60 years | [7-9] |
|  | Convergence amplitudes | ≥ 20 dpt | < 20 dpt | | | Fusion or no fusion |  |
| Ocular misalignment | Misalignment  Cover/uncover test | Normal alignment | Latent misalignment | Manifest misalignment | |  | Expert assessment |
| **Intra-ocular disorders** |  | **None** | **Mild** | **Moderate** | **Severe** |  |  |
| Cataract | LOCSIII  Nuclear colour/opalescence (NO) | NO 1-2 | NO 3 | NO 4 | NO 5-6 | Lens opacities classification system (LOCS III) or pseudophakia, or presence of lens capsule opacification | [10, 11] |
|  | LOCSIII Cortical (C ) | C 1 | C 2 | C 3 | C 4-5 |  |  |
|  | LOCSIII Posterior (subcapsular) (P) | P 1 | P 2 | P 3 | P 4-5 |  |  |
| Vitreous haemorrhage | Slit lamp examination | Not present | x | Present | |  | Expert assessment |
| **Optic nerve /retina disorders** |  | **None** | **Mild** | **Moderate** | **Severe** |  |  |
| Visual field deficit | Humphrey visual field analyser** | MD > 0 | MD -0.01 to -6 | MD -6.01 to - 12 | MD ≤ -12.01 | Mills et al Staging system, glaucoma severity staging system (GSS)  *With consideration of probability plot  * Excluding hemianopsia | [12] |
|  | Octopus visual field analyser* | MD ≤ -0.8 | MD -0.7 to 4.4 | MD 4.5 t0 9.5 | MD ≥15.4 | *With consideration of probability plot  MD: mean deviation | [13] |
| Intra ocular pressure | Tonometry Goldman | ≤ 18mmhg | 18-21mmhg | 21≥mmhg | |  | [14] |
| Cup-to-disc ratio  (CDR) | Fundus photography | ≤0.5 | >0.5 | >0.8 | | * small disc at risk with any CDR  * asymmetry >0.2 | [15, 16] |
| Glaucoma | Cup to disc ratio (>0.5)  Visual field deficit | Not present | x | Present | | *With consideration of IOP, amblyopia and cornea thickness | [17] |
| Colour vision | Ishihara | Normal | x | Abnormal | | Congenital colour blindness (red/green axis) | [18] |
|  | Farnsworth desaturated 15D Hue | None | Slight | Moderate | Severe | Green axis, red axis and blue axis | [19] |
| Contrast vision | Pelli robson | ≥2.00 | 1.50 -1.64 | 1.00-1.49 | <1.00 | Contrast sensitivity score (CSS)  1.65-1.95 normal for age 60-75 | [20, 21] |
| Maculopathy | Fundus picture / slit lamp examination | Not present | x | Present | | Scar, epiretinal gliosis, telangiectasia, macular oedema | Expert assessment |
| Age related macular degeneration | Fundus picture | No/normal changes | | Dry AMD | wet AMD |  | [22] |
| Retinopathy | Fundus picture  Slit lamp examination | Not present | Degeneration  Choroidal nevus | branch retinal vein occlusion, blot bleeding, surface wrinkling retinopathy, diabetic retinopathy, hypertensive retinopathy, | |  | Expert assessment |
| Peripheral retina drusen | Fundus picture  Slit lamp examination | Not present | x | Present | |  | Expert assessment |
| Vascular changes retina | Fundus picture | Not present | x | Present | |  | Expert assessment |
| Optic nerve neuropathy | Fundus picture  Slit lamp examination | Not present | Pallor of the optic nerve head, Tilted disc | Drusen of the optic nerve head  Atrophy of the optic nerve head | | small disc | Expert assessment |
| Ocular disease potential vision threatening | Severe dry eyes, cornea disease, retinopathy (branch retinal vein occlusion, blot bleeding, surface wrinkling retinopathy), optic neuropathy (optic nerve head drusen, optic nerve head atrophy, optic nerve head pallor), maculopathy (AMD, macula scar, epiretinal gliosis, telangiectasia, macular oedema), cataract (moderate/severe), PCO, vitreous haemorrhage, glaucoma | | | | | |  |
| Ocular disease clinically relevant | Visual hallucinations, dry eyes moderate/severe, convergence insufficiency moderate/severe, conjunctival disorder, eye lids disorder, cornea disease, misalignment manifest, retinopathy (branch retinal vein occlusion, blot bleeding, surface wrinkling retinopathy), optic neuropathy (optic nerve head drusen, optic nerve head atrophy, optic nerve head pallor) , maculopathy (AMD, macula scar, epiretinal gliosis, telangiectasia, macular oedema), cataract (moderate/severe), Lens capsule opacification (PCO), vitreous haemorrhage, glaucoma, homonymous hemianopia | | | | | |  |
| Ocular disease Yes | Visual hallucinations, dry eyes, convergence insufficiency, conjunctival disorder, eye lids disorder, cornea disease, misalignment manifest and latent, retinopathy (all), choroidal nevus, optic neuropathy (all) , maculopathy (AMD, macula scar, epiretinal gliosis, telangiectasia, macular oedema), cataract (moderate/severe), drusen peripheral, Lens capsule opacification, vitreous haemorrhage, glaucoma, homonymous hemianopia, congenital colour blind | | | | | |  |
| Ocular disease potential treatable | glaucoma, dry eyes, convergence insufficiency, ocular misalignment, cataract, vitreous haemorrhage, conjunctivitis, eye lids disorder, Lens capsule opacification, cornea disease. | | | | | |  |

AMD: Age related macular degeneration, MD: mean deviation, CSS: contrast sensitivity score, GSS: glaucoma severity staging system ,LOCSIII: Lens opacities classification system, NO: nuclear opalescence, NC: nuclear colour , P: posterior, C: cortical CDR: cup-to-disc ratio, EDTRS: Early Treatment of Diabetic Retinopathy Study, dpt: dioptre

*Expert assessment: evaluated and scored by experienced optometrist or ophthalmologist

** The mean deviation or mean defect (MD) gives an overall value of the total amount of visual field loss, with normal values typically within 0dB to -2dB. The MD value becomes more negative as the overall field worsens—a common example is when a cataract progresses. The use for tracking changes to localized field loss (as in glaucoma) is limited

**References**

1. Wong, T.Y. and P. Mitchell, *Hypertensive retinopathy.* N Engl J Med, 2004. **351**(22): p. 2310-7.

2. Stevens, G.A., et al., *Global prevalence of vision impairment and blindness: magnitude and temporal trends, 1990-2010.* Ophthalmology, 2013. **120**(12): p. 2377-84.

3. *The definition and classification of dry eye disease: report of the Definition and Classification Subcommittee of the International Dry Eye WorkShop (2007).* Ocul Surf, 2007. **5**(2): p. 75-92.

4. Behrens, A., et al., *Dysfunctional tear syndrome: a Delphi approach to treatment recommendations.* Cornea, 2006. **25**(8): p. 900-7.

5. Tamer, C., et al., *Tear film tests in Parkinson's disease patients.* Ophthalmology, 2005. **112**(10): p. 1795.

6. Milner, M.S., et al., *Dysfunctional tear syndrome: dry eye disease and associated tear film disorders - new strategies for diagnosis and treatment.* Curr Opin Ophthalmol, 2017. **27 Suppl 1**: p. 3-47.

7. Shaunak, S., E. O'Sullivan, and C. Kennard, *Eye movements.* J Neurol Neurosurg Psychiatry, 1995. **59**(2): p. 115-25.

8. Danchaivijitr, C. and C. Kennard, *Diplopia and eye movement disorders.* Journal of Neurology, Neurosurgery &amp; Psychiatry, 2004. **75**(suppl 4): p. iv24-iv31.

9. Convergence Insufficiency Treatment Trial Study, G., *Randomized clinical trial of treatments for symptomatic convergence insufficiency in children.* Arch Ophthalmol, 2008. **126**(10): p. 1336-49.

10. Chylack, L.T., Jr., et al., *Lens opacities classification system II (LOCS II).* Arch Ophthalmol, 1989. **107**(7): p. 991-7.

11. Chylack, L.T., Jr., et al., *The Lens Opacities Classification System III. The Longitudinal Study of Cataract Study Group.* Arch Ophthalmol, 1993. **111**(6): p. 831-6.

12. Susanna, R., Jr. and R.M. Vessani, *Staging glaucoma patient: why and how?* Open Ophthalmol J, 2009. **3**: p. 59-64.

13. Mills, R.P., et al., *Categorizing the stage of glaucoma from pre-diagnosis to end-stage disease.* Am J Ophthalmol, 2006. **141**(1): p. 24-30.

14. Chang, R.T. and K. Singh, *Glaucoma Suspect: Diagnosis and Management.* Asia Pac J Ophthalmol (Phila), 2016. **5**(1): p. 32-7.

15. Piette, S.D. and R.C. Sergott, *Pathological optic-disc cupping.* Curr Opin Ophthalmol, 2006. **17**(1): p. 1-6.

16. *European Glaucoma Society Terminology and Guidelines for Glaucoma, 4th Edition - Chapter 3: Treatment principles and options Supported by the EGS Foundation: Part 1: Foreword; Introduction; Glossary; Chapter 3 Treatment principles and options.* Br J Ophthalmol, 2017. **101**(6): p. 130-195.

17. *European Glaucoma Society Terminology and Guidelines for Glaucoma, 4th Edition - Chapter 2: Classification and terminologySupported by the EGS Foundation: Part 1: Foreword; Introduction; Glossary; Chapter 2 Classification and Terminology.* Br J Ophthalmol, 2017. **101**(5): p. 73-127.

18. Gorrell, G.J., *A study of defective colour vision with the Ishihara test plates.* Ann Hum Genet, 1967. **31**(1): p. 39-43.

19. Farnsworth, D., *Testing for color deficiency in industry.* AMA Arch Ind Health, 1957. **16**(2): p. 100-3.

20. Parede, T.R., et al., *Quality of vision in refractive and cataract surgery, indirect measurers: review article.* Arq Bras Oftalmol, 2013. **76**(6): p. 386-90.

21. Mantyjarvi, M. and T. Laitinen, *Normal values for the Pelli-Robson contrast sensitivity test.* J Cataract Refract Surg, 2001. **27**(2): p. 261-6.

22. Ferris, F.L., 3rd, et al., *Clinical classification of age-related macular degeneration.* Ophthalmology, 2013. **120**(4): p. 844-51.
